# Emergence of Linezolid Resistance Genes *optrA* and *cfr*(D) in an *Enterococcus saccharolyticus* from Chicken

**DOI:** 10.3390/antibiotics14040337

**Published:** 2025-03-24

**Authors:** Xun Gao, Xiao Luo, Ruorou Qian, Guolong Gao, Jinghao Liu, Junhao Hong, Chao Yue, Jian-Hua Liu, Yi-Yun Liu

**Affiliations:** 1 State Key Laboratory for Animal Disease Control and Prevention, College of Veterinary Medicine, South China Agricultural University, Guangzhou 510642, China; gxun@stu.scau.edu.cn (X.G.); 20223073092@stu.scau.edu.cn (X.L.); jchien@stu.scau.edu.cn (R.Q.); ggl@stu.scau.edu.cn (G.G.); 20223073073@stu.scau.edu.cn (J.L.); 202220110106@stu.scau.edu.cn (J.H.); yc_2023@stu.scau.edu.cn (C.Y.); 2 Key Laboratory of Zoonosis of Ministry of Agricultural and Rural Affairs, Guangzhou 510642, China

**Keywords:** antimicrobial resistance, linezolid, *optrA*, *cfr*(D), *Enterococcus saccharolyticus*

## Abstract

**Background:** The emergence of linezolid resistance, mediated by genes such as *optrA* and *cfr*(D), poses a growing public health threat. While these genes have been detected in clinical and animal-derived *Enterococcus* species, their presence in underexplored species like *Enterococcus saccharolyticus* remains undocumented, leaving a significant gap in our understanding of their dissemination and stability. **Method**: *E. saccharolyticus* GXN23C125Es was screened for the presence of known linezolid resistance genes via PCR analysis. Conjugation and stability experiments were used to evaluate the transferability and stability of the resistance genes. The complete genome of GXN23C125Es was obtained using both the Illumina and Nanopore platforms. **Results**: We report the first identification of *optrA* and *cfr*(D) in GXN23C125Es from chicken feces in China. Whole-genome sequencing revealed multiple plasmid-borne resistance genes, including *optrA*, *cfr*(D), *fexA,* and *erm*(A). Stability testing demonstrated that *optrA* was highly stable, while *cfr*(D) was rapidly lost without selective pressure. **Conclusions**: These findings highlight *E. saccharolyticus* as a potential reservoir for linezolid resistance genes, underscoring the need for enhanced surveillance of resistance determinants in animal-associated bacteria. Understanding the dissemination dynamics of *optrA* and *cfr*(D) is crucial for mitigating their impact on public health and guiding antimicrobial resistance management strategies.

## 1. Introduction

Linezolid, the first clinically approved oxazolidinone antibiotic, represents a critical last-resort treatment for severe infections caused by multidrug-resistant (MDR) Gram-positive pathogens, including vancomycin-resistant *Enterococcus* species (VRE), methicillin-resistant *Staphylococcus* species, and *Streptococcus pneumoniae* [1]. Since its introduction, linezolid has been highly effective in managing infections associated with hospital-acquired pathogens that exhibit resistance to other antimicrobial classes [2]. However, in recent years, the increasing prevalence of linezolid resistance among *Enterococcus* isolates worldwide has raised significant concerns regarding the sustainability of oxazolidinone-based therapy [3,4].

Linezolid resistance in Enterococcus is primarily mediated by three major mechanisms: (i) mutations in the 23S rRNA gene, particularly at position G2576T, which reduce the binding affinity of linezolid to the ribosomal peptidyl transferase center [4]; (ii) acquisition of transferable resistance genes, such as *cfr*, *cfr*(D), *optrA*, and *poxtA* [5]; and (iii) mutations in ribosomal protein-coding genes, such as *rplC*, *rplD*, and *rplV* [6]. Among these, the cfr and its variant genes encode a 23S rRNA methyltransferase that confers a multidrug resistance phenotype known as the PhLOPSA phenotype, which includes resistance to phenicols, lincosamides, oxazolidinones, pleuromutilins, and streptogramin A [2]. Conversely, the *optrA* and *poxtA* genes, both members of the ABC-F protein family, mediate resistance through ribosomal protection, reducing susceptibility to oxazolidinones and phenicols [7,8].

While linezolid has not been approved for use in food-producing animals, the emergence of linezolid-resistant *Enterococcus* isolates in pigs, poultry, and animal-derived food products suggests the widespread dissemination of resistance genes in agricultural settings [9,10,11]. Notably, the *optrA* gene was first identified in *E. faecalis* and *Enterococcus faecium* from both humans and food-producing animals in China [7], with the prevalence in animals steadily increasing over the years [3,12]. Additionally, the *optrA* and *cfr*(D) genes were first detected in a clinical *E. faecium* isolate in France [13,14], and, in recent years, these genes have also been reported to increase in animal populations [2,3]. Moreover, recent studies have documented the co-occurrence of multiple linezolid resistance genes on the same plasmid or within the same bacterial isolate, significantly enhancing the risk of horizontal gene transfer [15].

Here, we report, for the first time, the detection of the *optrA* and *cfr*(D) genes in *Enterococcus saccharolyticus* and provide a comprehensive genomic characterization of the *E. saccharolyticus* strain GXN23C125Es. This study aims to elucidate the genetic context, mobile elements, and potential transmission pathways of these resistance determinants, contributing to a broader understanding of antimicrobial resistance mechanisms and their implications for public health.

## 2. Results and Discussions

The *E. saccharolyticus* strain GXN23C125Es was isolated from chicken feces during a 2023 surveillance program aimed at identifying linezolid-resistant Gram-positive isolates. Antimicrobial susceptibility testing revealed that GXN23C125Es exhibited resistance to linezolid, gentamicin, tetracycline, florfenicol, erythrocin, and enrofloxacin, while remaining susceptible to penicillin and rifampin, but susceptible to tedizolid, a new oxazolidinone (Table 1). The extensive resistance profile of GXN23C125Es suggests the acquisition of multiple antimicrobial resistance determinants, posing significant concerns regarding its potential for horizontal gene transfer and persistence in diverse ecological niches. Whole genome sequencing (WGS) revealed that GXN23C125Es harbored multiple antimicrobial resistance genes, including *aac(6*′*)-aph(2*″*)*, *ant(6)-Ia* and *aph(3*′*)-III* (aminoglycoside resistance), cat (chloramphenicol resistance), *lsa*(E) and *lnu*(B) (lincosamides resistance), *erm*(B) and *erm*(A) (macrolide resistance), *dfrG* (trimethoprim resistance), *optrA* and *cfr*(D) (linezolid resistance), and *fexA* (chloramphenicol and florfenicol resistance). *E. saccharolyticus* is mainly found in environmental niches and the intestinal microbiota of animals and humans [16]. It has also been isolated from the milk of cows with mastitis [17]. However, few studies have focused on the antimicrobial resistance characteristics of this species, and this is the first report of the identification of *optrA* and *cfr*(D)-positive strains in *E. saccharolyticus* strains.

The assembly of both short and long sequencing reads produced a complete genome of approximately 2.68 Mb, comprising a 2,639,213 bp chromosome and five plasmids ranging from 9447 to 41,280 bp in size. All identified antimicrobial resistance genes were plasmid-borne. Detailed analysis showed that *cfr*(D) was located on pHNGX23C125Es-3, which also carried *optrA*, *fexA*, and *erm*(A) (Table 1), and sequence analysis revealed that the plasmid could not be typed. Conjugation experiments showed that pHNGX23C125Es-3 could not be transferred to recipient strains, and sequence analysis confirmed the absence of conjugative transfer regions on this plasmid, explaining its non-conjugative nature.

**Figure 1:**
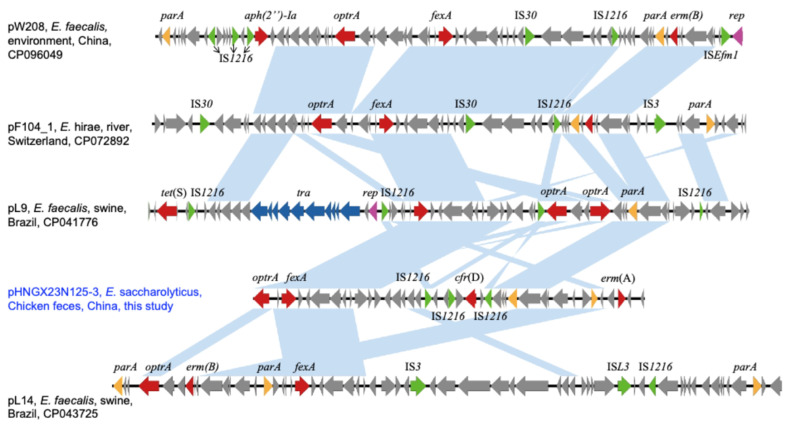
Comparison of pHNGX23N125-3 with similar structures isolated from different strains and sources. Purple, red, green, yellow, blue and gray arrows represent replication genes, antimicrobial resistance genes, mobile element genes, participation system genes, conjugative transfer genes and hypothetical protein genes, respectively. Light blue shading denotes regions of shared 99% homology among the different structures.

BLASTn analysis identified four plasmids in the GenBank database with over 55% coverage and 99.5% sequence identity to pHNGX23C125Es-3. Among these, plasmids pL14 (67% coverage, 98.76% identity, CP043725) and pL9 (60% coverage, 98.76% identity, CP041776), carried by *E. faecalis* strains L14 and L9 isolated from swine in Brazil, showed the highest similarity. Comparative analysis indicated that the ~80 kb variable region harboring *fexA* was conserved among these plasmids. Notably, although *optrA* and *fexA* were located on the same plasmid, unlike the previously described Tn6674-associated structures [18], no direct genetic linkage was observed between these genes in pHNGX23C125Es-3 and its similar plasmids (Figure 1). The genetic environment surrounding *cfr*(D) consisted of the structure IS*1216-hp*-*apt*-IS*1216-guaA-cfr*(D)*-hp*-IS*1216*. Previous studies have shown that *cfr*(D) often coexists with IS*1216* [19], which can mediate its mobility by forming transposable units with varying structures [20]. These findings suggest that IS*1216* may play a pivotal role in the dissemination of *cfr*(D).

To evaluate the stability of *optrA* and *cfr*(D), we conducted serial passaging of the GXN23C125Es strain in antibiotic-free Luria–Bertani (LB) broth. The *optrA* gene exhibited 100% stability in the absence of antibiotic pressure after 7 days (approximately 70 generations) in the natural host GXN23C125Es. In contrast, the *cfr*(D) gene was rapidly lost, with only 50% retention observed on the first day, and complete loss by the fifth day (Figure S1). These findings indicate that, in the absence of selective pressure, *optrA* is highly stable within the host, whereas *cfr*(D) demonstrates significantly lower stability. The differential stability of *optrA* and *cfr*(D) may be attributed to variations in their genetic contexts. For example, *cfr*(D) may be excised via IS*1216*-mediated recombination [21], resulting in reduced stability in the host strain. Alternatively, differences in the fitness cost imposed by *optrA* and *cfr*(D) on the host bacterium may play a role, as resistance genes can impose varying degrees of metabolic burden on their bacterial hosts [22], thereby affecting their persistence. Further studies are required to elucidate the precise mechanisms underlying these observations.

The detection of linezolid-resistant *E. saccharolyticus* in poultry feces raises significant public health concerns, particularly given the increasing prevalence of oxazolidinone resistance in enterococci isolated from food animals [23]. The potential for zoonotic transmission of linezolid-resistant strains is particularly alarming, as previous studies have reported the presence of *optrA*- and *cfr*(D)-harboring enterococci in retail meat, animal-derived food products, and even companion animal diets [24]. Furthermore, the persistence of linezolid resistance genes in environmental reservoirs, such as livestock manure and wastewater, poses additional risks for the dissemination of resistance through horizontal gene transfer [23,25]. Given the widespread use of florfenicol in veterinary medicine, co-selection of oxazolidinone resistance genes may be inadvertently facilitated by the use of phenicol antibiotics, further exacerbating the spread of resistance [2,4].

## 3. Conclusions

This study presents the first report of the whole genome sequence of a *cfr*(D)-carrying *Enterococcus saccharolyticus* strain, GXN23C125Es, isolated from chicken feces. The plasmid harboring *cfr*(D) also carried *optrA*, *fexA*, and *erm*(A), conferring resistance to multiple clinically important antibiotics. The presence of Gram-positive bacteria harboring multiple resistance genes poses a significant public health risk. These findings highlight the urgent need for continued surveillance of the prevalence and dissemination of *optrA*, *cfr*(D), and their carriers.

## 4. Materials and Methods

### 4.1. Strain Isolation and Detection of Linezolid Resistance Genes

*E. saccharolyticus* strain GXN23C125Es was isolated from the feces of a chicken during our surveillance program focused on detecting linezolid-resistant Gram-positive isolates in 2023. A total of 240 chicken fecal samples were collected using swabs from a poultry farm in Guangxi, China. The samples were transferred to 5 mL of Luria–Bertani (LB) medium and enriched at 37 °C. Following enrichment, 100 μL of each sample was transferred to a subculture in 5 mL of fresh LB medium containing 5% NaCl and 10 mg/L florfenicol, and incubated for 24 h. Subsequently, 20 μL of the enriched culture was streaked onto Columbia agar supplemented with 5% (*v*/*v*) sheep blood and 10 mg/L florfenicol and incubated at 37 °C for 24 h. Colonies exhibiting target morphology were selected and subcultured on fresh selective media for purification. Species identification was confirmed by MALDI-TOF MS (Bruker Daltonik GmbH, Bremen, Germany). The presence of resistance genes *optrA*, *poxtA*, *cfr*, *cfr*(B), *cfr*(C), and *cfr*(D)was determined by PCR and Sanger sequencing, using primers listed in Table S1.

### 4.2. Antimicrobial Susceptibility Testing

Minimum inhibitory concentrations (MICs) of antimicrobial agents for *E. saccharolyticus* strain GXN23C125Es were determined by the broth microdilution method according to Clinical and Laboratory Standards Institute (CLSI) guidelines [26,27]. The following antibiotics were tested: linezolid, tedizolid, chloramphenicol, florfenicol, erythromycin, tetracycline, penicillin G, ciprofloxacin, and vancomycin. Susceptibility was interpreted based on CLSI recommendations. *E. faecalis* ATCC 29212 and *Staphylococcus aureus* ATCC 29213 were used as quality control strains.

### 4.3. Whole Genome Sequencing (WGS) and Genome Analysis

Genomic DNA from *E. saccharolyticus* GXN23C125Es was subjected to whole-genome sequencing using both short-read Illumina NovaSeq 6000 (San Diego, CA, USA) and hybrid long-read sequencing (Oxford Nanopore MinION, Oxford, UK). De novo assembly of the short- and long-read data was performed using Unicycler v0.4.4 [28]. The antimicrobial resistance genes and sequence type of the assembled scaffolds were identified using ResFinder v4.1 and MLST v2.0, respectively, at the Center for Genomic Epidemiology (https://www.genomicepidemiology.org/). The assembled genomes were annotated using the RAST server (https://rast.nmpdr.org/) [29], and BLASTN was performed to compare plasmid sequences with known sequences from the NCBI database (https://blast.ncbi.nlm.nih.gov/Blast.cgi).

### 4.4. Conjugation Experiments

The transferability of plasmids carrying linezolid resistance genes was evaluated by conjugation experiments. *E. faecalis* JH2-2 was used as the recipient strain. Putative transconjugants were selected on brain heart infusion agar plates supplemented with 10 mg/L florfenicol and 100 mg/L rifampicin. The transconjugants were identified by MALDI-TOF MS and screened for resistance genes using PCR. Conjugation frequency was calculated as the number of transconjugants divided by the number of recipient cells.

### 4.5. Plasmid Stability Testing

The stability of *optrA* and *cfr*(D) in the host strain was evaluated by passaging in antibiotic-free LB broth. Three single clones from each *optrA* and *cfr*(D)-positive strain were cultured overnight in 3 mL of LB without antibiotics at 37 °C. The cultures were then subcultured daily by diluting 1:100 in fresh LB broth for seven consecutive days. At the end of each day, 100 colonies were randomly selected from antibiotic-free MacConkey agar plates, and the presence of *optrA* and *cfr*(D) was confirmed by PCR (Table S1). The plasmid retention rate was calculated as the percentage of colonies harboring *optrA* and *cfr*(D) out of the 100 colonies tested.

### 4.6. Accession Numbers

The genome sequences of *E. saccharolyticus* strain GXN23C125Es have been deposited in the GenBank database under the accession numbers CP180195-CP180200.

## Figures and Tables

**Table 1:** Characterization of GXN23C125Es.

Strain	Location	Size (bp)	Plasmid Type	Drug Resistance Genes	MIC (μg/mL)							
					GEN	ERY	TET	FLOR	ENR	VAN	LIN	TED
GXN23C125Es	chromosome	2,639,213			16	>256	64	>128	8	4	8	0.5
	pHNGXN23C125-1	41,280	repUS1-repE(DOp2)	*aac(6*′*)-aph(2*″*), cat, ant(6)-Ia, lsa*(E)*, lnu*(B)*, aph(3*′*)-III, erm*(B)								
	pHNGXN23C125-2	38,208	repUS1-rep(pVEF1)	*dfrG, erm*(B)								
	pHNGXN23C125-3	37,500	/	*optrA, fexA, cfr*(D)*, erm*(A)								
	pHNGXN23C125-4	33,200	/									
	pHNGXN23C125-5	9447	/									

GEN, gentamicin; ERY, erythrocin; TET, tetracycline; FLOR, florfenicol; ENR, enrofloxacin; VAN, vancomycin; LIN, linezolid; TED, tedizolid.

## Data Availability

The data presented in this study are available upon request.

## Supplementary Materials

The following supporting information can be downloaded at: https://www.mdpi.com/article/10.3390/antibiotics14040337/s1, Table S1: Primers used in this study; Figure S1: Stability of *optrA* and *cfr*(D) genes in the corresponding host isolate. References [5,7,30] are cited in the Supplementary Materials.
