# Supplementary material for: Emergence of Linezolid Resistance Genes optrA and cfr(D) in an Enterococcus saccharolyticus from Chicken
Source: Antibiotics (Basel). 2025 Mar 24;14(4):337. doi: 10.3390/antibiotics14040337 (PMC12024316; doi:10.3390/antibiotics14040337)
Supplement: Supplementary file 1 [file antibiotics-14-00337-s001.zip › antibiotics-3488265-supplementary.pdf]

Supplementary Material

Table S1 Primers used in this study

**Primer**	**Sequence (5'-3')**	**Size (bp)**	**Annealing temperature**	**Reference**
optrA-F	AGGTGGTCAGCGAACTAA	1395	54℃	[1]
optrA-R	ATCAACTGTTCCCATTCA			
poxtA-F	GAACGCTTGGAGTATTTCGACTTC	778	54℃	[2]
poxtA-R	CTGGACTGAGAATACCCATC			
cfr-F	TGAAGTATAAAGCAGGTTGGGAGTCA	746	50℃	[3]
cfr-R	ACCATATAATTGACCACAAGCAGC			
cfr(B)-F	CAGGAGACGAAAAAATAGAAAC	398	50℃	[2]
cfr(B)-R	AAAAGGAGAATGTAATGAAAATGT			
cfr(C)-F	GGTGAAACTGTTGTGGAGAT	474	50℃	[2]
cfr(C)-R	AGTAGGTGCGTGGAGGGAATA			
cfr(D)-F	TGGCTGGGAATCTTTTTGTA	304	50℃	[2]
cfr(D)-R	TAGTCGTTTTATTTTAGGAA			

Figure S1 Stability of *optrA* and *cfr(D)* genes in the corresponding host isolate.

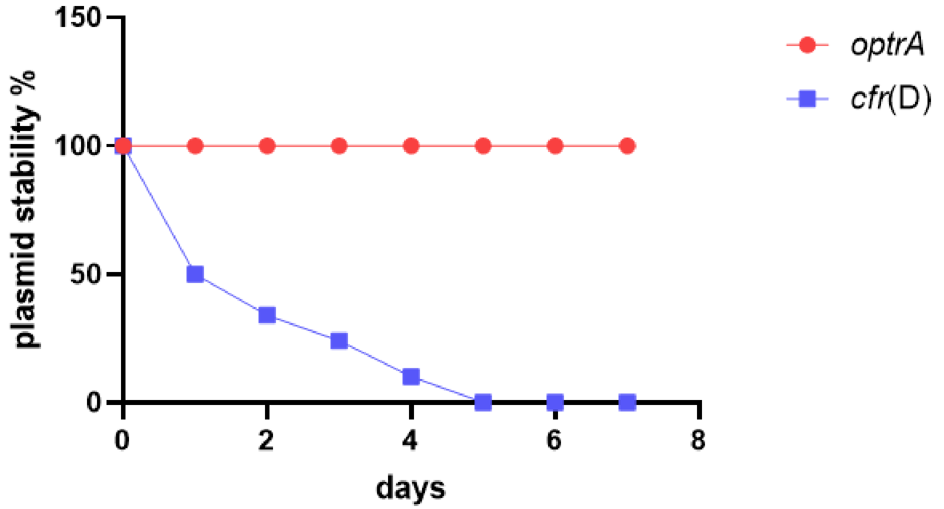

1. Wang, Y.; Lv, Y.; Cai, J.; Schwarz, S.; Cui, L.; Hu, Z.; Zhang, R.; Li, J.; Zhao, Q.; He, T.; et al. A novel gene, *optrA*, that confers transferable resistance to oxazolidinones and phenicols and its presence in *Enterococcus faecalis* and *Enterococcus faecium* of human and animal origin. *J Antimicrob Chemother* **2015**, *70*, 2182-2190.
2. Cinthi, M.; Coccitto, S.N.; Fioriti, S.; Morroni, G.; Simoni, S.; Vignaroli, C.; Magistrali, C.F.; Albini, E.; Brenciani, A.; Giovanetti, E. Occurrence of a plasmid co-carrying *cfr(D)* and *poxtA2* linezolid resistance genes in *Enterococcus faecalis* and *Enterococcus casseliflavus* from porcine manure, Italy. *J Antimicrob Chemother* **2022**, *77*, 598-603.
3. McHugh, M.P.; Parcell, B.J.; Pettigrew, K.A.; Toner, G.; Khatamzas, E.; El Sakka, N.; Karcher, A.M.; Walker, J.; Weir, R.; Meunier, D.; et al. Presence of *optrA*-mediated linezolid resistance in multiple lineages and plasmids of *Enterococcus faecalis* revealed by long read sequencing. *Microbiology (Reading)* **2022**, *168*.
